# Protective Effect of *Lycium ruthenicum* Polyphenols on Oxidative Stress against Acrylamide Induced Liver Injury in Rats

**DOI:** 10.3390/molecules27134100

**Published:** 2022-06-25

**Authors:** Hua Gao, Yanzhong Xue, Lingyu Wu, Jinghong Huo, Yufei Pang, Jingxin Chen, Qinghan Gao

**Affiliations:** 1 Department of Pharmacy, General Hospital of Ningxia Medical University, Yinchuan 750004, China; gaohualike@163.com; 2 School of Public Health and Management, Ningxia Medical University, Yinchuan 750004, China; Yanzhongxue1997@163.com (Y.X.); wulingyu50155@163.com (L.W.); huojinghun@163.com (J.H.); PYF157356420342021@163.com (Y.P.); chenjx_96@163.com (J.C.); 3 Key Laboratory of Environmental Factors and Chronic Disease Control, Ningxia Medical University, Yinchuan 750004, China

**Keywords:** acrylamide, hepatotoxicity, *Lycium ruthenicum*, mitochondrial function, oxidative stress

## Abstract

Acrylamide (ACR) is formed during tobacco and carbohydrate-rich food heating and is widely applied in many industries, with a range of toxic effects. The antioxidant properties of *Lycium ruthenicum* polyphenols (LRP) have been established before. This study aimed to research the protective effect of LRP against ACR-induced liver injury in SD rats. Rats were divided into six groups: Control, ACR (40 mg/kg/day, i.g.), LRP (50, 100, and 200 mg/kg/day, i.g.) plus ACR, and LRP groups. After 19 days, we evaluated oxidative status and mitochondrial functions in the rat’s liver. The results showed that glutathione (GSH) and superoxide dismutase (SOD) levels increased after LRP pretreatment. In contrast, each intervention group reduced reactive oxygen species (ROS) and malondialdehyde (MDA) levels compared to the ACR group. Meanwhile, alanine aminotransferase (ALT), aspartate aminotransferase (AST), liver mitochondrial ATPase activity, mRNA expression of mitochondrial complex I, III, and expression of nuclear factor-erythroid 2-related factor 2 (Nrf2) and its downstream proteins were all increased. This study suggested that LRP could reduce ACR-induced liver injury through potent antioxidant activity. LRP is recommended as oxidative stress reliever against hepatotoxicity.

## 1. Introduction

Acrylamide (ACR) is widely used to synthesize industrial chemicals such as polyacrylamide [1]. Previous studies have found that ACR has severe harmful effects on mammals’ genetic material, nervous system, and immune function [2,3,4,5,6]. Furthermore, ACR can be exposed to humans in several ways and threaten human health [1,7,8]. In 2002, Swedish researchers found high-concentration ACR in starchy foods after heat treatment [9]. Since then, ACR has become a foodborne hazard, attracting the attention of researchers all over the world [7]. With the changing dietary habits and the increasing consumption frequency of baked and fried foods, people can intake a certain amount of ACR through their daily diet, resulting in higher exposure and increasingly serious hazards for their health [10]. In addition, children and young people are especially easily exposed to foods containing ACR, such as French fries, bread, cookies, and so on [1,11,12].

As the primary metabolic target organ of ACR, the liver is the first to be affected [13]. Aspartate aminotransferase (AST) and alanine aminotransferase (ALT) are closely related to liver function [14]. Previous studies have shown that ACR can increase the serum levels of ALT and AST and inhibit the activation of alkaline phosphatase (ALP) in mice [15]. Oxidative damage and mitochondrial dysfunction have been identified as the main mechanisms of ACR hepatotoxicity [16]. Excessive reactive oxygen species (ROS) production can lead to oxidative stress. ROS can destroy cellular macromolecules and lead to apoptosis [17,18,19]. It has been shown that the expression levels of antioxidant enzymes were reduced in the livers of SD rats after 21 days of ACR intervention, such as glutathione (GSH), superoxide dismutase (SOD), and catalase. In contrast, total oxidative status and malondialdehyde (MDA) levels were increased [6,20]. In addition, a study has shown that mitochondrial dysfunction is inextricably linked to the production of large amounts of ROS. The mitochondrial membrane potential of hepatocytes was altered under ACR induction [21]. Nuclear factor-erythroid 2-related factor 2 (Nrf2) is released from Keap1 after ROS activation. It will bind to the nucleus’s antioxidant response element (ARE), and increased the expression of peroxidase, such as heme oxygenase-1 (HO-1), glutamate-cysteine ligase catalase (GCLC), glutamate-cysteine ligase (GCLM), and quinone oxidoreductase 1 (NQO1) [22]. ACR treatment could also altered the expression of Nrf2 [23]. From the above information, it is easy to see the potential association mechanism among oxidative stress, mitochondrial damage, and hepatotoxicity of ACR. Nowadays research on ACR has been mainly focused on minimizing its production during food production process. Little research was carried out on reducing the oxidative damage caused by the unavoidable daily intake of ACR with natural antioxidants. Based on the fact that liver is the important site of ACR metabolism, this study aimed to focus on whether natural antioxidant such as polyphenols widely available in vegetables and fruits can ameliorate ACR-induced hepatotoxicity.

Polyphenols are natural antioxidants with good antioxidant properties, which can significantly inhibit oxidative stress and inhibit the occurrence of mitochondrial dysfunction. *Lycium ruthenicum* (LR), a well-known perennial plant in the Solanaceae family, found throughout the Qinghai-Tibet Plateau. It has been used as a traditional herb to cure heart illness, menstrual disorders, vision disorders, and always as an antihypertensive medicine. The bioactivity of LR has recently been discovered to have antioxidant, antihyperlipidemic, and anti-inflammatory properties. *Lycium ruthenicum* polyphenols (LRP) are an essential active ingredient of the LR [24]. Our previous studies have described the characterization and quantification of LRP by HPLC in detail. The main phenolic compounds in LRP are rutin, p-coumaric acid, catechin, and caffeic acid [25]. LRP as antioxidants could minimize ROS production and prevent lipid peroxidation in vitro and in vivo [25,26,27]. Meanwhile, this polyphenol has certain hepatoprotective effect [28]. Previous studies have focused on the antioxidant activity and cultivation of LR. However, the antihepatotoxic activity and mechanism of LRP are still unclear.

Therefore, based on the wide exposure of ACR and its strong hepatotoxicity, the present study aimed to investigate the protective effects and mechanism of LRP against ACR-induced hepatic oxidative and mitochondrial function damage in rats. In detail, we observed the morphology and ultrastructure of rat liver, and determined serum ALT, AST, oxidative stress indexes, liver mitochondrial ATP activity and mitochondrial complex I, II, III mRNA expression.

## 2. Results

### 2.1. Effect of LRP on the ACR-Induced Serum Levels of ALT and AST

Our results displayed marked elevations in the serum levels of ALT and AST (*p* < 0.01) in ACR-intoxicated rats than in control (CON) rats. LRP supplementation at different doses in the ACR-intoxicated group markedly reduces (*p* < 0.01) the serum levels of ALT and AST compared to the ACR-intoxicated group (Table 1).

### 2.2. Effect of LRP on the ACR-Induced Histopathological Changes

**Figure 1:**
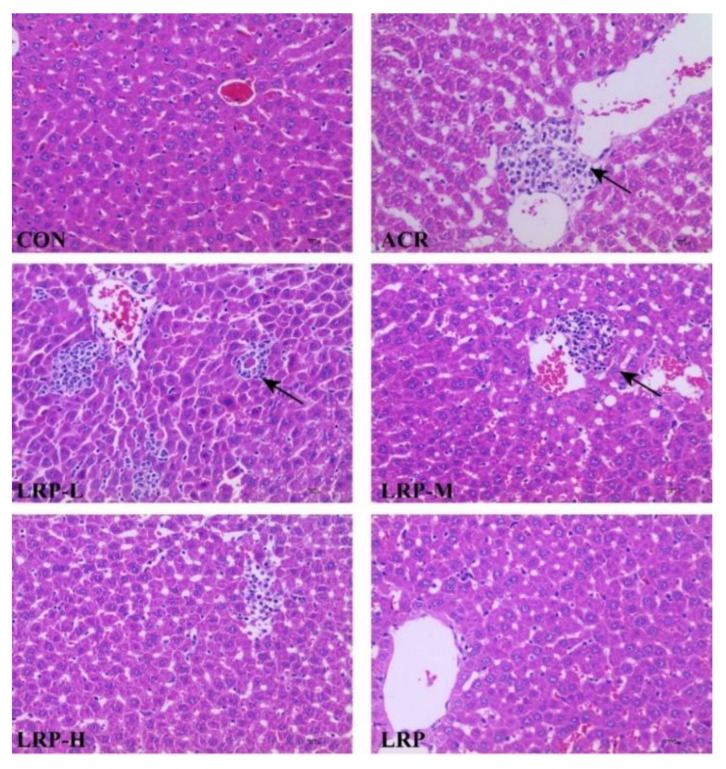
Effect of LRP on the ACR-induced histopathological changes (hematoxylin and eosin (H&E), 400×, bar 10 μm). The black arrow, inflammatory cell infiltration. ACR, acrylamide; LRP-L, *Lycium ruthenicum* polyphenols in low dosage; LRP-M, *Lycium ruthenicum* polyphenols in medium dosage; LRP-H, *Lycium ruthenicum* polyphenols in high dosage; LRP, *Lycium ruthenicum* polyphenols control group.

Hepatic tissue from the CON and LRP groups were with typical histological structures under light microscopy. The liver from the ACR group showed localized inflammatory cell infiltration and some damage to hepatocytes. Three doses of LRP pretreated with LRP-L, LRP-M, and LRP-H slightly improved the histopathological morphology induced by ACR (Figure 1).

**Figure 2:**
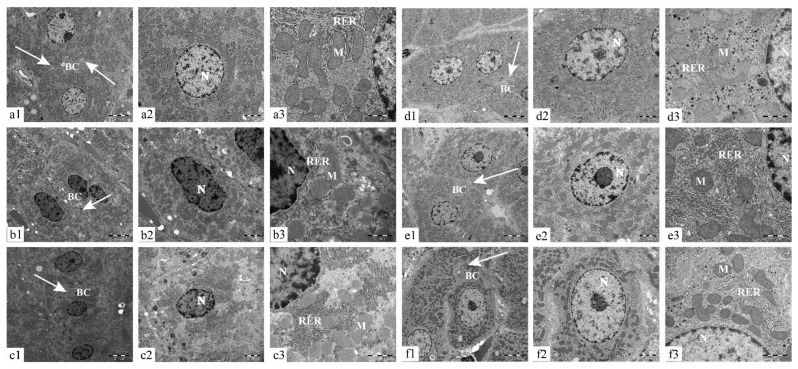
Effect of LRP on the ultrastructure of ACR-induced liver tissue in SD rats (1-5000×, 2-10000×, 3-30000×), CON, ACR, LRP-L, LRP-M, LRP-H, LRP groups are expressed with (**a**–**f**), respectively. Abbreviations: N-nucleus; RER- rough endoplasmic reticulum; M- mitochondria; BC- bile canaliculus; arrows, junctional complex. ACR, acrylamide; LRP-L, *Lycium ruthenicum* polyphenols in low dosage; LRP-M, *Lycium ruthenicum* polyphenols in medium dosage; LRP-H, *Lycium ruthenicum* polyphenols in high dosage; LRP, *Lycium ruthenicum* polyphenols control group.

Transmission electron microscopy showed that the hepatocytes in the CON and LRP groups had regular morphology with clear borders, mitochondria were abundant, and the endoplasmic reticulum’s surface was rough; the cristae of mitochondria had a clear structure. Compared to the CON group, hepatocytes in the ACR group suffered from organelle destruction, mitochondrial swelling, mitochondrial cristae loss, and rough endoplasmic reticulum. After the intervention of three doses of LRP, the nuclear structure of hepatocytes gradually improved, and the mitochondrial membrane structure and rough endoplasmic reticulum were restored with does dependant (Figure 2).

### 2.3. Effects of LRP on ROS in the Liver

**Figure 3:**
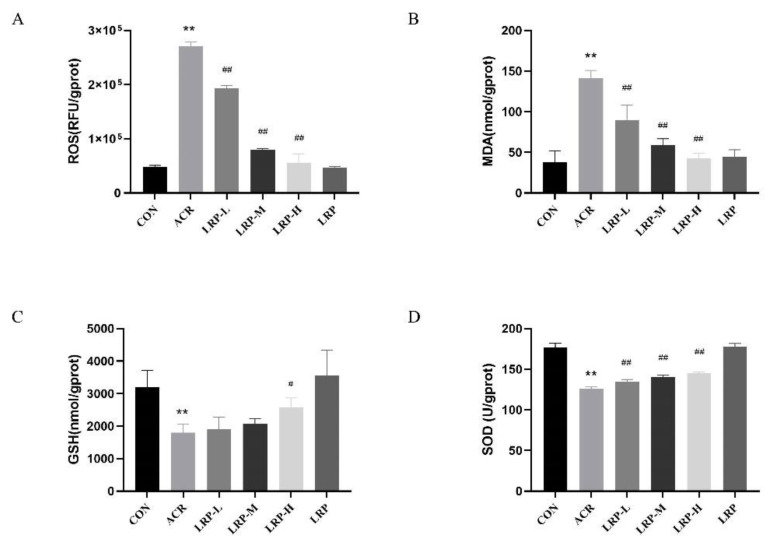
Effects of LRP on ROS (**A**), MDA (**B**), GSH (**C**), and SOD (**D**) in the liver. Data are expressed as mean ± SD. ** *p* < 0.01 versus the CON group; ^#^ *p* < 0.05 and ^##^ *p* < 0.01 versus the ACR group. ACR, acrylamide; LRP-L, *Lycium ruthenicum* polyphenols in low dosage; LRP-M, *Lycium ruthenicum* polyphenols in medium dosage; LRP-H, *Lycium ruthenicum* polyphenols in high dosage; LRP, *Lycium ruthenicum* polyphenols control group.

As one can see in Figure 3A, ACR (40 mg/kg) significantly increased the level of hepatic ROS after 12 days of continuous intervention (*p* < 0.01). In addition, the ROS were decreased in the LRP (50, 100, and 200 mg/kg) groups compared to the ACR group (*p* < 0.01).

### 2.4. Effects of LRP on SOD, GSH, and MDA in the Liver

As can be see in Figure 3, administration of ACR (40 mg/kg) for 12 consecutive days significantly increased the MDA level compared to the control group in the liver (*p* < 0.01). ACR-treated rats showed lower levels of SOD and GSH than the CON group (*p* < 0.01). In contrast, different doses of LRP inhibited oxidative damage in liver tissue. In addition, the levels of MDA were significantly decreased in the LRP (50, 100, 200 mg/kg) group compared to the ACR group rats (*p* < 0.01). Compared with the ACR group, administration with LRP at 50, 100, and 200 mg/kg dose increased the levels of SOD and GSH (*p* < 0.05).

### 2.5. Effect of LRP on ATPase Activities Induced by ACR in the Liver Mitochondrion

As shown in Table 2, liver mitochondrial ATPase activity was significantly decreased in ACR-treated rats compared with the CON group (*p* < 0.01). In addition, Na^+^/K^+^-ATPase activity and Mg^2+^-ATPase activity in the ACR group were approximately 50% lower than those in the CON group. LRP pretreatment significantly restored the activity of these ATPases, and at the high dose (200 mg/kg) of LRP essentially restored the Ca^2+^-ATPase activity to control levels (*p* < 0.05).

### 2.6. Effect of LRP on the mRNA Expression of Mitochondrial Complexes I–III Induced by ACR in Liver Tissue

**Figure 4:**
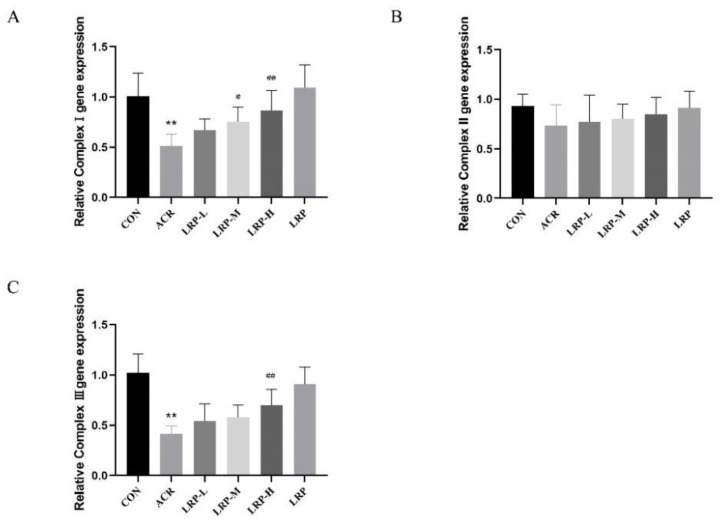
Effect of LRP on the mRNA expression of mitochondrial complexes I (**A**), II (**B**), III (**C**) induced by ACR in liver tissue. Data are expressed as mean ± SD. ** *p* < 0.01 versus the CON group; ^#^ *p* < 0.05 and ^##^ *p* < 0.01 versus the ACR group. ACR, acrylamide; LRP-L, *Lycium ruthenicum* polyphenols in low dosage; LRP-M, *Lycium ruthenicum* polyphenols in medium dosage; LRP-H, *Lycium ruthenicum* polyphenols in high dosage; LRP, *Lycium ruthenicum* polyphenols control group.

Compared with the CON group, we did not find any significant change in complex II in the ACR group, but a significant decline was observed in mRNA levels of complexes I and III (*p* < 0.01). Furthermore, administration of LRP at the doses of 50, 100, and 200 mg/kg in ACR-induced toxicity rats, significantly increased mRNA levels of complexes I and III compared with the ACR group (*p* < 0.01) (Figure 4).

### 2.7. Effect of LRP on the Nrf2 Pathway Induced by ACR in Liver Tissue

**Figure 5:**
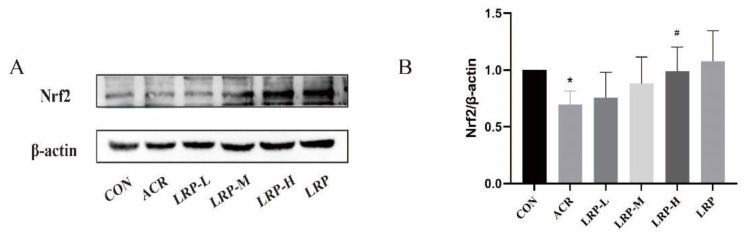
(**A**) Effect of LRP on Nrf2 expression in the liver. The protein expression analysis from the liver of an experimental rat using Nrf2 antibodies. (**B**) The data of the densitometric analysis of Nrf2/β-actin. Data are expressed as mean ± SD. * *p* < 0.05 versus the CON group; ^#^ *p* < 0.05 versus the ACR group. ACR, acrylamide; LRP-L, *Lycium ruthenicum* polyphenols in low dosage; LRP-M, *Lycium ruthenicum* polyphenols in medium dosage; LRP-H, *Lycium ruthenicum* polyphenols in high dosage; LRP, *Lycium ruthenicum* polyphenols control group.

Compared with the CON group, Nrf2, NQO1, GCLC, GCLM, and HO-1 proteins expression was reduced significantly in the ACR group (*p* < 0.01). ACR can reduce intracellular Nrf2-regulated downstream antioxidant proteins Nrf2, NQO1, GCLC, GCLM, and HO-1. In contrast, the expression levels of antioxidant proteins in the Nrf2 pathway, including Nrf2, NQO1, GCLC, GCLM, and HO-1 were increased after LRP pretreatment with a dose-response effect. These results suggest that LRP may exert antioxidant effects by regulating the expression of Nrf2 pathway antioxidant proteins (Figure 5 andFigure 6).

## 3. Discussion

The aim of this study was to investigate the protective effect of LRP against ACR-induced hepatotoxicity. ACR, a water-soluble toxic substance with solid permeability, can quickly go into the bloodstream and may trigger liver damage [29]. Recently, a study by Elhelaly et al. indicated that ACR-induced toxicity caused damage to liver tissue by increasing DNA oxidative damage and reducing antioxidant enzyme activity [30]. Previously, we reported that LRP had a protective impact on oxidative damage in vitro. In this study, we further investigated the protective effect of LRP against ACR-induced toxicity in rat liver and its related mechanism.

The liver is the target organ for exogenous toxicants, which is considerably more sensitive to these toxicants and plays a vital role in the detoxification process [4]. One previous study showed ACR-induced oxidative stress in the liver by allicin compared with the CON group. The levels of ALT and AST were increased in the ACR group mice, indicating the ACR staining has caused acute damage to liver function. The vitality of ALT and AST in all groups of allicin has decreased with the increase of allicin concentration, and the liver function was also improved [31]. The results we obtained were consistent with those that have been reported. The activity of ALT and AST in the serum of rats in the ACR group was significantly higher, indicating that the liver function was damaged by the rupture of hepatocytes and even mitochondria. There were significant differences in the ALT and AST viability in each dose group of LRP compared with the ACR group. The LRP improved the recovery of membrane structure and normalized the permeability and function of hepatocytes under ACR-induced liver injury. Therefore, LRP has a protective effect on ACR-induced liver function injury in rats. It has been shown that the central veins, sinus venosus, and vascular structures in the portal area of the liver profile are congested after ACR administration. Inflammatory cells infiltrate the portal connective tissue and periportal area. Areas of hepatocyte necrosis and hemorrhage of different sizes were seen in the liver parenchyma [17,30]. From the results of this experiment, it was observed that there was local inflammatory cell infiltration in the liver of the ACR group, and the degree of the damage was not significant. In order to observe some changes that occurred in the liver, we also observed the liver ultrastructure with transmission electron microscopy. The results showed that the nuclear structure of hepatocytes was damaged after the action of ACR, the mitochondrial bilayer structure was not clear, and the rough endoplasmic reticulum was proliferated. The above changes in the ultrastructure of hepatocytes mean that the liver is damaged [32]. From this study, we can know that LRP can promote the recovery of hepatocyte membrane structure after ACR-induced liver injury and restore its permeability and function to normal in rats. It indicates that LRP has a protective effect on ACR-induced liver injury in rats with dose-dependence.

Oxidative stress occurs in an imbalance between the production and elimination of oxidative products in the body when stimulated by various external harmful substances. A previous study showed that ACR could disrupt the balance between oxidation and antioxidants. It is linked with the overproduction of ROS and thus leads to oxidative damage [19,33]. Our current study suggested ACR-induced oxidative stress in rat tissues. SOD and GSH activities were significantly reduced. Conversely, the level of MDA and ROS increased during ACR treatment. Our results were consistent with others that ACR could increase lipid peroxidation and cause damage to the antioxidant enzyme systems [29]. Different studies suggested the antioxidant effects of LR [34,35]. Tian et al. found that LRP normalizes high lipid peroxidation levels in mice [36]. In addition, the GSH content of the rodent liver was significantly increased after gavage LRP. In the present study, all three doses of LRP (50, 100, and 200 mg/kg) reduced MDA levels, while different amounts of LRP were effective in increasing the levels of GSH in the liver tissue.

Mitochondria are the main sites of endogenous ROS generation and easily occur oxidative stress. The results showed that ACR reduced the activity of ATP hydrolase, which affected mitochondrial function and resulted in insufficient energy supply in vivo. It is consistent with Er et al. [37]. In addition, we observed mitochondrial structural damage in ACR-induced hepatocytes by transmission electron microscopy. We found that LRP can directly scavenge the free radicals generated during mitochondrial damage. Polyphenols restore mitochondrial ATPase activity by maintaining the structural and functional integrity of the inner and outer mitochondrial membranes. Zhao et al. [38] found that blueberry anthocyanins restored ACR-induced Na^+^, K^+^-ATPase, and Mg^2+^-ATPase activities in rats with simultaneous dose-response effects. Our results indicate that LRP increases mitochondrial ATPase activity in a dose-dependent manner. Compared with the ACR group, the activity of ATPase in the LRP group was significantly increased. Complexes I, II, III, and IV have electron transport functions, and complexes I and III are necessary sites for ROS production. The change in its activity can reflect the change in mitochondrial respiratory function. Er et al. observed that the mitochondrial respiratory enzyme activity (complexes I–V) and the phospholipid level in the center of the mitochondrial membrane decreased after ACR induction in mouse liver [37]. We found LRP could significantly reduce ACR-induced mRNA expression of mitochondrial complexes I and III genes in rat liver. No differences were found for complex II mRNA. ACR inhibits the function of the mitochondrial respiratory chain complex and reduces its activity, leading to a decrease in ATP synthesis [39]. The antioxidant properties of LRP can slow down the damage of ACR to the mitochondrial complex, thus maintaining the normal electron transport function in the mitochondrial respiratory transport chain. The results showed that LRP medium and high doses could inhibit the decrease of complexes I and III mRNA expression. According to previous studies [38], blueberry anthocyanin extract can inhibit electron leakage of complexes I and III induced by ACR, thus improving mitochondrial function in the liver.

Nrf2/ antioxidant response element (ARE) is an important antioxidant signaling pathway. In addition to Nrf2 nuclear translocation, the Nrf2 pathway is mainly responsible for ARE activation. Through Nrf2 activation, downstream targets such as HO-1, GCLM, GCLC, and NQO1 are highly expressed [40]. Previous experiments have shown that the Nrf2 signaling pathway is a crucial compensatory protective mechanism that attenuates ACR-induced oxidative damage [41,42]. Our results showed that ACR induced a decrease in Nrf2, HO-1, GCLC, GCLM, and NQO1 in the liver of rats compared to the CON group. Liu et al. found that chlorogenic acid could be protective in rats with cerebral ischemia/reperfusion injury by activating the Nrf2 pathway [43]. Tan et al. suggested that ACR-treated cells activated the Nrf2/NQO-1 pathway and increased the expression of mitochondrial respiratory complexes by resveratrol in ACR-treated cells [44]. This study also confirmed that LRP has a potent antioxidant activity to increase Nrf2, HO-1, GCLC, GCLM, and NQO1 protein expression in a dose-dependent manner. Based on the above results, it is further demonstrated that LRP can alleviate the damage of mitochondrial structure and function and inhibit apoptosis through its good antioxidant effect, thus playing a role in protecting ACR-induced hepatotoxicity. This study provides a new perspective to improve the hepatotoxicity induced by ACR intake with LRP.

## 4. Materials and Methods

### 4.1. Chemicals and Reagents

ACR was purchased from Sigma-Aldrich (St. Louis, MO, USA). Tris-HCl, EDTA-Na2, and sucrose were purchased from Biotopped (Beijing, China). GSH, AST, ALT, MDA, SOD, Bradford protein assay kit, and Mitochondrial ATPase were bought from Nanjing Jiancheng Bioengineering Institute (Nanjing, China). PVDF membranes were purchased from Merck Millipore (Burlington, MA, USA). Anti-Nrf2 antibodies were purchased from Proteintech (Wuhan, China). Anti-HO-1, anti-NQO1, anti-GCLC, anti-GCLM, and anti-β-actin were obtained from Abcam (Cambridge, UK). Trizol, PrimeScript™ RT Master Mix wi, and TB Green Premix Ex Taq™ II were purchased from Takara (Kusatsu, Japan). All chemicals employed in this work were analytical grade. The LRP sample preparation was according to the method of Gao et al. [25]. LRP was composed of nine phenolic compounds, among which rutin was the most abundant at 1013.05 ± 33.70 mg/100 g, followed by p-coumaric acid, catechin, caffeic acid [25].

### 4.2. Animals and Experimental Design

Forty-eight healthy male Spraque Dawley rats (7-week old and weighing 225–275 g) were obtained from the Experimental Animal Center of Ningxia Medical University (Yinchuan, China). The study was carried out in agreement with the Ethical Committee Acts and Guidelines of Ningxia Medical University of Medical Sciences (ethical number: IACUC-NYLAC-2020-119). Rats were randomly divided into six groups, including the control (CON) group, ACR group, three LRP intervention groups in low, medium, and high dosages (LRP-L, LRP-M, LRP-H), and LRP control group (LRP group), each group consisting of 8 animals. These groups are shown in Table 3. After one week of adaptive feeding, rats in the LRP intervention group were gavaged with the corresponding dose of LRP for 7 days, while rats in the control and ACR groups were gavaged with normal saline. On the eighth day of the intervention, rats were gavaged with ACR in each group. Meanwhile, the corresponding dose of LRP or regular saline for 1 h before manipulation for 12 days. The dose settings of ACR and LRP were obtained from pre-experiment and related literature [19,45].

### 4.3. Sample Collection

By the end of the experimental time, isoflurane was used for rat anesthesia. After the corneal reflex, flip reflex, and pain reflex disappeared, the liver tissue was removed by exposing the abdomen. The blood on the surface of the tissue was washed with saline and placed in a lyophilization tube. After that, the treated tissues were quickly cryopreserved in liquid nitrogen and stored at −80 °C.

### 4.4. Isolation of Liver Mitochondria

We extracted rat liver mitochondria with reference to the method of Zhang et al. [38]. Briefly, about 0.1 g of rat liver tissue was taken, washed, homogenated, and centrifuged at 600× *g* for 10 min. Prepared the supernatant for further use was taken, centrifuged at 11,000× *g* for 15 min, and precipitated as mitochondria. The prepared mitochondria were stored at −80 °C for the future.

### 4.5. Histopathological Studies

Liver tissue was fixed in 10% formalin, processed in wax blocks, sliced, dyed, closed, etc., and examined under a microscope [15].

### 4.6. Transmission Electron Microscopy

Liver tissue was fixed in 2% glutaraldehyde for 4 h at 4 °C and rinsed 3 times with 0.1 M phosphate buffer at 2 h intervals; soaked in 1% osmium acid for 2 h, and rinsed twice with 0.1 M phosphate buffer for 15 min each; dehydrated in 30% ethanol for 10 min; dehydrated in 50% ethanol for 10 min; dehydrated in 70% ethanol for 10 min; died in 80% ethanol dehydration, 10 min; 90% ethanol dehydration, 10 min; 100% ethanol dehydration, 15 min, 2 times; propylene oxide infiltration, 15 min × 2 times; 1:1 (incomplete embedding solution: propylene oxide) infiltration, 1 h; 2:1 (incomplete embedding solution: propylene oxide) infiltration, one h; insufficient embedding solution infiltration, overnight. Complete embedding solution immersion, 35 °C constant temperature, 6 h. Under the transmission electron microscope, transfer to the embedding plate (complete embedding solution) at 42 °C overnight and under observation for 48 h at 60 °C [46].

### 4.7. Measurement of the AST, ALT Indexes in Serum

The enzymes AST and ALT activities were determined with the commercial assay kits referring to the manufacturers’ instructions [47].

### 4.8. Assessment of Oxidative Stress in the Liver

The absorbance values were measured at 450 nm (SOD), 412 nm (GSH), and 532 nm (MDA) for SOD, GSH, and MDA, respectively [48].

The liver single-cell suspension was prepared, and DCFH-DA was added at a recommended concentration of 10 µM. The cells were incubated at 37 °C for 30 min. The probe labeled single-cell suspension was collected, washed once or twice with PBS, centrifuged, and collected the precipitate. The fluorescence intensity was measured at the optimal excitation wavelength of 485 nm and the optimal emission wavelength of 525 nm. The measurement results were expressed as fluorescence intensity/mg protein.

### 4.9. Measurement of the Liver Mitochondrial ATPase

The activities of Na^+^-K^+^-ATPase, Ca^2+^-ATPase, and Mg^2+^-ATPase were detected using a commercial kit [49].

### 4.10. Total RNA Extraction, Reverse Transcription, and Quantitative Real-Time Polymerase Chain Reaction

The tissue RNA was extracted by the Trizol method, the RNA concentration was measured, and the extracted total RNA was reverse transcribed to cDNA according to the reverse transcription kit instructions. Quantitative real-time PCR analysis was performed on the PCR system using TB Green Premix Ex Taq™ II. Then relative gene expression was normalized to GAPDH and calculated using the ^ΔΔ^CT method [50]. The primer sequences utilized in the research are listed in Table 4.

### 4.11. Western Blot Analysis

Samples were mixed with 6× supersampling buffer and denatured at 100 °C for 5 min. Equal amounts of total proteins were electrophoresed using SDS-PAGE and then transferred to PVDF membranes (Merck Millipore, Immobilon-P, Burlington, MA, USA) using a transblot device. PVDF membranes were blocked in 5% skim milk PBST buffer. Then they were incubated with rabbit primary antibodies Nrf2 (1:1000), HO-1 (1:10,000), NQO1 (1:10,000), GCLC (1:20,000), GCLM (1:1000), and β-actin (1:5000) at 4 °C overnight. After washing, the cells were incubated with rabbit or mouse horseradish peroxidase-conjugated anti-IgG (1:4000) for 1 h at room temperature. Finally, protein bands were observed with the ECL reagent. Protein density analysis was performed using Alliance Image J software. All protein bands were standardized to β-actin [51].

### 4.12. Statistical Analysis

Results were expressed as mean ± SD. All statistical comparisons were performed by a one-way ANOVA test followed by Tukey’s post hoc analysis. SPSS (version 22.0, IBM, Armonk, NY, USA) and GraphPad Prism 6.0 were used for drawing and data analysis. *p* < 0.05 was considered significant.

## 5. Conclusions

The present study showed that ACR could induce liver injury through oxidative stress and mitochondrial dysfunction. Moreover, oral LRP administration at 50, 100, and 200 mg/kg/day could protect the rat’s liver tissue from ACR-induced oxidative stress in a dose-dependent manner, improving mitochondrial structure and function by activating Nrf2 signaling pathway. It is suggested that LRP could be a promising liver protective agent against ACR toxicity. LRP is recommended as oxidative stress relievers against hepatotoxicity.

## Figures and Tables

**Figure 6:**
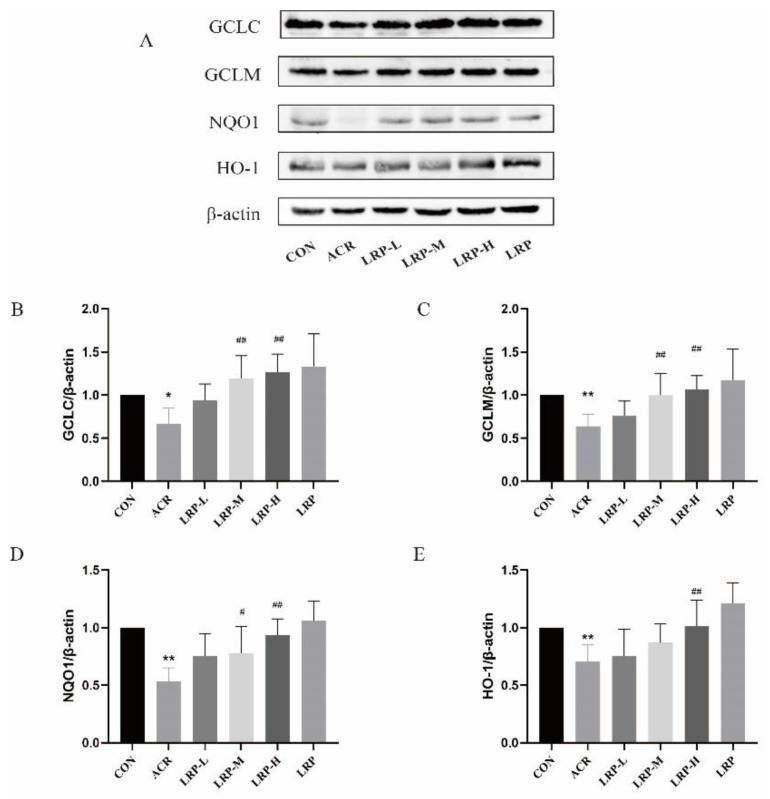
(**A**) Effect of LRP on the expression of HO-1, GCLC, GCLM, and NQO1 in the liver. The protein expression analysis from the liver of an experimental rat using HO-1, GCLC, GCLM, and NQO1 antibodies. The data of the densitometric analysis of (**B**) GCLC/β-actin, (**C**) GCLM/β-actin, (**D**) NQO1/β-actin, (**E**) HO-1/β-actin. Data are expressed as mean ± SD. * *p* < 0.05 and ** *p* < 0.01 versus the CON group; ^#^ *p* < 0.05 and ^##^ *p* < 0.01 versus the ACR group. ACR, acrylamide; LRP-L, *Lycium ruthenicum* polyphenols in low dosage; LRP-M, *Lycium ruthenicum* polyphenols in medium dosage; LRP-H, *Lycium ruthenicum* polyphenols in high dosage; LRP, *Lycium ruthenicum* polyphenols control group.

**Table 1:** Effect of LRP on the ACR-induced serum levels of ALT and AST.

Groups/ Parameters	CON	ACR	LRP-L	LRP-M	LRP-H	LRP
ALT (U/L)	60.08 ± 9.22	124.18 ± 19.31 **	82.03 ± 15.76 ^#^	92.48 ± 22.17 ^#^	77.36 ± 26.19 ^#^	67.48 ± 6.03
AST (U/L)	100.45 ± 9.81	204.60 ± 33.93 **	165.19 ± 23.04 ^#^	160.52 ± 21.71 ^#^	146.51 ± 22.21 ^##^	122.99 ± 10.36

Note: Data are expressed as the mean ± SD. ** *p* < 0.01 versus the CON group; ^#^ *p* < 0.05 and ^##^ *p* < 0.01 versus the ACR group. ACR, acrylamide; ALT, alanine transferase; AST, aspartate transferase; LRP-L, *Lycium ruthenicum* polyphenols in low dosage; LRP-M, *Lycium ruthenicum* polyphenols in medium dosage; LRP-H, Lycium ruthenicum polyphenols in high dosage; LRP, *Lycium ruthenicum* polyphenols control group.

**Table 2:** The Effect of LRP on the level of ATPase activities in liver mitochondrial.

Groups/ Parameters	CON	ACR	LRP-L	LRP-M	LRP-H	LRP
Ca^2+^-ATPase	2.28 ± 0.56	1.39 ± 0.17 **	1.60 ± 0.11	1.64 ± 0.16	1.90 ± 0.46 ^#^	2.31 ± 0.54
Na^+^-K^+^-ATPase	2.50 ± 0.49	1.40 ± 0.23 **	1.57 ± 0.14	1.76 ± 0.42	1.97 ± 0.45	2.48 ± 0.69
Mg^2+^-ATPase	2.43 ± 0.50	1.40 ± 0.22 **	1.61 ± 0.10	1.71 ± 0.41	1.92 ± 0.45	2.39 ± 0.57
Total-ATPase	7.29 ± 1.55	4.26 ± 0.63 **	4.78 ± 0.34	5.07 ± 0.98	5.89 ± 1.48	7.35 ± 1.64

Note: Data are expressed as the mean ± SD. ** *p* < 0.01 versus the CON group; ^#^ *p* < 0.05 versus the ACR group. ACR, acrylamide; LRP-L, *Lycium ruthenicum* polyphenols in low dosage; LRP-M, *Lycium ruthenicum* polyphenols in medium dosage; LRP-H, *Lycium ruthenicum* polyphenols in high dosage; LRP, *Lycium ruthenicum* polyphenols control group.

**Table 3:** Study design.

Groups	0–7 d Treatment (i.g.)	8–19 d Treatment (i.g.)
CON	normal saline	normal saline
ACR	normal saline	normal saline + ACR(40 mg/kg)
LRP-L	LRP(50 mg/kg)	LRP(50 mg/kg) + ACR(40 mg/kg)
LRP-M	LRP(100 mg/kg)	LRP(100 mg/kg) + ACR(40 mg/kg)
LRP-H	LRP(200 mg/kg)	LRP(200 mg/kg) + ACR(40 mg/kg)
LRP	LRP(200 mg/kg)	LRP(200 mg/kg)

Note: ACR, acrylamide; LRP-L, *Lycium ruthenicum* polyphenols in low dosage; LRP-M, *Lycium ruthenicum* polyphenols in medium dosage; LRP-H, *Lycium ruthenicum* polyphenols in high dosage; LRP, *Lycium ruthenicum* polyphenols control group.

**Table 4:** Sequences of primers for quantitative real-time PCR.

Genes	Forward Primer	Reverse Primer
COX I	CTGCCCTCTGTACCCAAAGA	GACCCATCTTTCCAGAGGT
COX II	CCAGATGGCCAGAGGACTCA	TGTGAGTCCCGAGGGAATAGA
COX III	GCCACCACACCCCTATTGTA	TCCCGTTGCTATGAAGAATG
GAPDH	TGTTCCTACCCCCAATGTGT	CCCTGTTGCTGTAGCCGTAT

## Data Availability

Data is contained within the article.

## Sample Availability

Samples of the compounds are available from the authors.
